# Age at natural menopause, reproductive lifespan and Alzheimer’s disease in females: is APOE ε4 the missing link?

**DOI:** 10.3389/fgene.2026.1733593

**Published:** 2026-03-11

**Authors:** Francesco Bruno, Patrizia Spadafora, Paolo Abondio, Antonio Qualtieri, Ersilia Paparazzo, Mirella Aurora Aceto, Ida Veltri, Selene De Benedittis, Beatrice Maria Greco, Annamaria Cerantonio, Luigi Citrigno, Gemma Di Palma, Olivier Gallo, Giuseppe Passarino, Alberto Montesanto, Francesca Cavalcanti

**Affiliations:** 1 Department of Human and Social Sciences, Universitas Mercatorum, Rome, Italy; 2 Institute for Biomedical Research and Innovation (IRIB), Italian National Research Council (CNR), Cosenza, Italy; 3 Department of Biology, University of Rome Tor Vergata, Rome, Italy; 4 Department of Biology, Ecology and Earth Sciences, University of Calabria, Arcavacata di Rende, Italy; 5 Unit of Geriatric Medicine, Italian National Research Center on Aging - INRCA IRCCS, Cosenza, Italy; 6 Territorial Social-Health Company of Lodi, Lodi, Italy

**Keywords:** age at natural menopause, Alzheimer’s disease, APOE genotype, dementia risk, estrogen exposure, females, reproductive lifespan, sex differences

## Abstract

**Background:**

The apolipoprotein E (APOE) gene represents the strongest genetic determinant of sporadic Alzheimer’s disease (AD), yet its interaction with sex-specific endocrine factors remains poorly understood. Lifetime estrogen exposure, estimated through reproductive lifespan, may modulate neurodegenerative risk, but findings are inconsistent. Previous studies have examined reproductive factors and APOE interactions in relation to cognitive outcomes, but dose-dependent effects across all APOE alleles (ε2, ε3, ε4) in clinically diagnosed AD patients remain underexplored. This study investigates the joint effects of reproductive lifespan, age at natural menopause (ANM), and APOE genotype on AD risk in females.

**Methods:**

A total of 396 female participants (103 with AD, 293 cognitively healthy controls) were retrospectively analyzed. Demographic, clinical, and reproductive data were extracted from medical records. APOE genotyping was performed by sequencing rs429358 and rs7412 polymorphisms. Logistic regression models tested associations between ANM, reproductive lifespan, and AD diagnosis, adjusting for education, body mass index (BMI), smoking, diabetes, hypertension, and number of children. Moderation analyses assessed the interaction between reproductive variables and APOE ε2, ε3, and ε4 alleles, and were followed by simple slope analyses to clarify the direction of significant effects.

**Results:**

AD females exhibited later ANM (50.3 ± 4.4 vs. 48.3 ± 6.2 years; *p* = 0.004) and longer reproductive lifespan (37.4 ± 4.4 vs. 35.4 ± 6.0 years; *p* = 0.005) than controls. Both ANM and reproductive lifespan independently predicted higher AD risk (adjusted OR = 1.07, 95% CI = 1.02–1.12, *p* < 0.01). These effects were amplified by APOE ε4 and attenuated by ε3, while ε2 showed no influence. Simple slope analyses confirmed an allele-specific gradient, with the association between later menopause and AD risk steepest in ε4 carriers and absent in high ε3 carriers.

**Conclusion:**

This work provides novel evidence that extended ovarian function is associated with increased AD vulnerability in females, particularly among APOE ε4 carriers. These findings highlight a dose-dependent, genotype-specific interaction between reproductive aging and neurodegeneration, suggesting APOE as a molecular bridge linking estrogenic exposure and AD risk.

## 1 Introduction

Dementia represents one of the major public health challenges worldwide, with its burden steadily increasing due to population aging. Alzheimer’s disease (AD) is the most common form of dementia, accounting for 60%–70% of all cases (Cummings and Cole, 2002). Clinically, AD is characterized by progressive cognitive decline, particularly in memory and executive functions, and is frequently accompanied by neuropsychiatric symptoms such as depression and apathy (Altomari et al., 2022; Laganà et al., 2022). These manifestations contribute substantially to caregiver burden and accelerate disease progression. AD exists in both rare monogenic forms, caused by mutations in genes such as *APP*, *PSEN1*, and *PSEN2*, and in the far more prevalent sporadic forms (Bruno et al., 2023a). The latter are considered multifactorial, arising from the interplay between genetic susceptibility, environmental exposures, and endocrine-metabolic influences (Suresh et al., 2023; Bruno et al., 2022; Bruno et al., 2023b; Bruno et al., 2025a; Chakrabarti et al., 2015).

Among genetic factors, the apolipoprotein E (APOE) gene represents the strongest and most consistently replicated risk factor for sporadic AD. In particular, the ε4 allele has been associated with a substantially increased probability of developing AD and with an earlier age of onset (Corder et al., 1993; Farrer et al., 1997). Conversely, the ε2 allele has been described as potentially protective, while ε3 represents the most common and generally considered neutral variant (Abondio et al., 2023). However, genetic predisposition alone does not account for the wide clinical heterogeneity observed in AD, suggesting that additional environmental and endocrine factors contribute to modulating risk.

In females, reproductive history is a major determinant of lifetime exposure to estrogens, hormones that exert neuroprotective effects by enhancing synaptic plasticity, regulating glucose metabolism, and attenuating oxidative stress and neuroinflammation (Brinton, 2009). Estrogen exposure has therefore been hypothesized to partially explain sex differences in dementia prevalence and progression (Price et al., 2025). Epidemiological evidence, however, has produced conflicting results. Most prior research has focused on age at natural menopause (ANM) as a proxy for cumulative estrogen exposure. Several studies reported that later ANM is associated with better late-life cognitive outcomes (Kuh et al., 2018). However, ANM alone may not fully capture the complexity of reproductive aging, as it ignores the contribution of menarche and the overall duration of ovarian function. The concept of reproductive lifespan - defined as the interval between menarche and menopause - has therefore gained increasing attention as a more comprehensive indicator of cumulative estrogen exposure (Georgakis et al., 2016).

**FIGURE 1:**
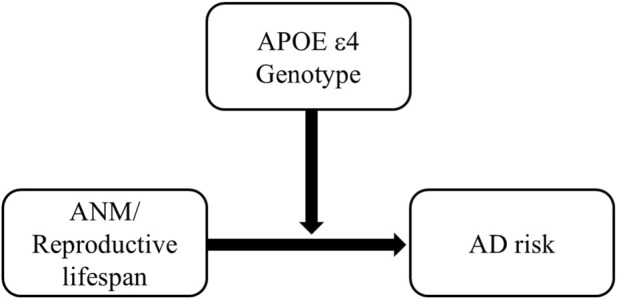
Conceptual model illustrating the hypothesized moderating role of the APOE ε4 genotype in the association between reproductive factors (age at natural menopause [ANM] and reproductive lifespan) and sporadic Alzheimer’s disease (AD) risk in females. The model assumes that the relationship between prolonged ovarian function and AD risk varies according to APOE ε4 allele dosage, with a stronger positive association expected among ε4 carriers. ANM = Age at Natural Menopause; AD = Alzheimer’s Disease.

Importantly, reproductive aging is not solely determined by environmental and lifestyle factors, but is also under strong genetic influence, with heritability estimates for ANM ranging between 49% and 63% (de Bruin et al., 2001; Murabito et al., 2005). Among genetic determinants, APOE has attracted particular interest. Beyond its role in lipid metabolism and AD pathophysiology, APOE variants appear to modulate reproductive traits. Evidence is, however, inconsistent: APOE ε4 carriers have been reported to experience later ANM compared to non-carriers in some cohorts (Meng et al., 2012), while other studies found no association (He et al., 2009) or even suggested earlier ANM in ε4-positive females in specific populations (Koochmeshgi et al., 2004). Such discrepancies likely reflect ethnic variability, differences in study design, and the frequent reliance on ANM as the sole reproductive marker. Taken together, these findings raise the hypothesis that the relationship between reproductive history and AD risk cannot be understood without considering APOE genotype. Specifically, APOE may act as a “missing link” connecting reproductive lifespan with dementia vulnerability, amplifying or buffering the effects of cumulative estrogen exposure depending on allelic status. The interaction between reproductive factors and APOE genotype in relation to cognitive outcomes has been previously investigated, though with inconsistent findings. Geerlings et al. (2001), in the Rotterdam study of 1,357 postmenopausal females, reported that longer reproductive period was associated with increased dementia risk, with this association being stronger among APOE ε4 carriers. More recently, Lindseth et al. (2023) examined over 221,000 middle-aged females from the United Kingdom Biobank and found that longer reproductive span was associated with better cognitive performance but reported no significant APOE ε4 interactions. Studies examining hormone replacement therapy have similarly yielded mixed results regarding APOE moderation (Yaffe et al., 2000; Kang and Grodstein, 2012). These inconsistencies likely reflect differences in study design, outcome measures (cognitive performance vs. dementia diagnosis), population characteristics, and the focus on APOE ε4 carrier status rather than dose-dependent effects across all alleles. Building upon this evidence, the present study aimed to investigate the association between ANM, reproductive lifespan and clinically diagnosed AD in females, while testing dose-dependent moderation effects across all three APOE alleles (ε2, ε3, and ε4) rather than focusing solely on ε4 carrier status. Particular attention was devoted to ε4, hypothesized to exacerbate the impact of reproductive lifespan on cognitive vulnerability, whereas ε2 and ε3 were examined in an exploratory fashion given their putative protective or neutral roles (Figure 1).

## 2 Materials and methods

### 2.1 Subjects

The study included Italian patients with sporadic AD, who were followed at the National Research Council (CNR) - Institute for Biomedical Research and Innovation (IRIB) in Mangone, Cosenza, Italy. All participants were of Caucasian/European ancestry. Data were retrospectively retrieved from their medical records, provided the clinical information was complete. The inclusion criteria were as follows: (i) age over 18 years; (ii) diagnosis of probable AD according to NINCDS-ADRDA criteria (McKhann et al., 1984) and National Institute on Aging and Alzheimer’s Association Workgroup (McKhann et al., 2011); (iii) absence of mutations in *APP*, *PS1*, and *PS2* genes; (iv) no history of head trauma, neurological conditions other than AD, intellectual disability, or serious medical conditions; (v) natural menopause (participants with surgical menopause due to hysterectomy and/or bilateral oophorectomy were excluded) and; (vi) availability of a blood sample. The following clinical and demographic variables were recorded for each patient: sex, age, years of education, body mass index (BMI), smoking status, presence of diabetes, hypertension, age at menarche, number of children, ANM, and reproductive lifespan (calculated as the interval between menarche and menopause). Reproductive variables (age at menarche, age at menopause, number of children) were based on self-reported information recorded at the time of clinical assessment, as documented in medical records and, when available, supported by caregiver reports or other clinical documentation. A group of cognitively healthy controls (CTRL) was also included. CTRL participants were recruited from the same geographic area and were matched to patients for year of birth through the Unical - University of Calabria (Rende, Cosenza, Italy). Inclusion criteria for CTRLs were: absence of neurological or psychiatric disorders, no history of major medical conditions affecting cognition. Demographic and clinical characteristics recorded for CTRLs included age, sex, years of education, body mass index (BMI), smoking status, and presence of diabetes or hypertension (for more details, see Corsonello et al., 2010; Montesanto et al., 2010). Year of birth was used to match participants on birth cohort, thereby controlling for generational differences in educational attainment, reproductive patterns, and life-course exposures. The research was conducted in accordance with the Helsinki Declaration of 1975. Ethical approval was not required, as the study utilized non-identifiable data and anonymized biological materials, in line with local regulations. Written informed consent was obtained from all participants for the blood sample collection and clinical data.

### 2.2 APOE genotyping

Genomic DNA was isolated from peripheral venous blood samples using the classical *Salting Out* method (Miller et al., 1988). Following the protocol previously described by Bruno et al. (2025b), a 318 bp fragment of exon 4 of the APOE gene was amplified by PCR using specific forward (5′-ACT​GAC​CCC​GGT​GGC​GGA​GGA​GAC​GCG​GGC-3′) and reverse (5′-TGT​TCC​ACC​AGG​GGC​CCC​AGG​CGC​TCG​CGG-3′) primers. APOE genotypes were then determined through direct sequencing of the rs429358 and rs7412 polymorphisms, employing the Big Dye Terminator v3.1 Cycle Sequencing Kit and an ABI PRISM 3130 XL Genetic Analyzer (Applied Biosystems, Life Technologies).

### 2.3 Statistical analysis

Descriptive statistics were computed for demographic, reproductive, and clinical characteristics. Means and standard deviations (M ± SD) were calculated for continuous variables, while frequencies and counts (n) were reported for categorical variables. Group differences were assessed with independent t-tests or Chi-Square (χ^2^)/Fisher’s exact tests as appropriate. Differences in the distribution of APOE genotypes and alleles between cognitively healthy controls (CTRL) and patients with Alzheimer’s disease (AD) were examined with contingency table analyses; column percentages were reported. APOE allele frequencies were derived from genotype data by counting each allele separately, assuming two alleles per individual. Fisher’s exact tests were applied to assess overall group differences. To test whether ANM or reproductive lifespan were associated with AD, binomial logistic regression analyses were conducted. In the unadjusted model (Model 1), ANM or reproductive lifespan (years) was entered as the sole predictor. In the adjusted model (Model 2), ANM or reproductive lifespan was entered together with a set of prespecified covariates: years of education, number of children, body mass index (BMI), smoking status (yes/no), diabetes (yes/no), and hypertension (yes/no). To examine whether the association between reproductive lifespan or ANM and AD diagnosis was moderated by APOE alleles, moderation analyses were performed using logistic regression models (Jamovi *medmod* module). In each model, ANM or reproductive lifespan (years) was entered as the independent variable, diagnostic status (CTRL vs. AD) as the dependent variable, and the count of each specific APOE allele (ε2, ε3, or ε4; range 0–2) as the moderator. Three separate moderation models were fitted, one for each allele type, to test allele-specific dose-dependent effects. For each participant, the moderator represented the number of copies of the specific allele being tested (0, 1, or 2 copies). For example, in the ε4 moderation model, the moderator took values of 0 (ε2/ε2, ε2/ε3, ε3/ε3 genotypes), 1 (ε2/ε4, ε3/ε4 genotypes), or 2 (ε4/ε4 genotype). The conditional effects of reproductive lifespan and ANM were estimated at low (−1 SD), mean, and high (+1 SD) levels of each moderator. Missing data were handled using listwise deletion. All analyses were performed using Jamovi software (version 2.3.18). A p < 0.05 was considered statistically significant.

## 3 Results

### 3.1 Demographic, reproductive and clinical characteristics of the sample

Table 1 summarizes the demographic, reproductive, and clinical characteristics of the study population. No significant differences were observed between AD patients and controls in year of birth, education, age at menarche, number of children, BMI, hypertension, diabetes, or smoking status (all *p* > 0.05). By contrast, AD patients showed a significantly later ANM (50.34 ± 4.36 vs. 48.35 ± 6.16 years, *p* = 0.004) and a longer reproductive lifespan (37.43 ± 4.40 vs. 35.40 ± 6.01 years, *p* = 0.005) compared with controls. These findings indicate that extended ovarian function characterized AD patients, whereas vascular and metabolic risk factors did not significantly differ between groups.

**TABLE 1:** Demographic, reproductive and clinical characteristics of the sample.

Variable	CTRL (n = 293)	AD (n = 103)	Total N	*p*-value
Demographic and reproductive				
Years of birth, mean	1931 ± 8.88	1930 ± 8.78	396	0.193
Education, <8 years	244 (83.3)	85 (82.5)	396	0.861
Age at menarche, years	13.18 ± 1.75	13.02 ± 1.75	372	0.442
Age at natural menopause, years	48.35 ± 6.16	50.34 ± 4.36	369	0.004**
Reproductive lifespan, years	35.40 ± 6.01	37.43 ± 4.40	345	0.005**
Children, n	3.27 ± 2.02	3.08 ± 1.75	381	0.411
Clinical				
BMI, kg/m^2^	27.73 ± 4.89	28.26 ± 4.36	373	0.181
Hypertension, n (%)	82 (49.7)	88 (43.6)	372	0.241
Diabetes, n (%)	87 (27.0)	16 (22.5)	393	0.437
Smoking, n (%)	9 (2.8)	5 (7.0)	390	0.086

Values are expressed as *mean ± SD*, for continuous variables and *n (%)* for categorical variables. *p*-values are from independent *t*-tests (continuous variables) or χ^2^ test (categorical variables), with Fisher’s exact test applied when expected cell counts were <5. CTRL, cognitively healthy controls; AD, Alzheimer’s disease patients. BMI, body mass index. Total N reflects the number of participants with available data for each variable. *p < 0.05; **p < 0.01.

### 3.2 Distribution of APOE genotypes and alleles in CTRL and AD groups

The distribution of APOE genotypes significantly differed between cognitively healthy controls and AD patients (χ^2^ = 57.2, df = 5, *p* < 0.001). As shown in Table 2, the ε3/ε3 genotype was predominant in both groups, but its frequency was substantially higher in controls compared to AD patients (78.9% vs. 52.0%). Conversely, AD patients showed a strong enrichment of ε3/ε4 (40.8% vs. 7.9%) and ε4/ε4 (2.0% vs. 0.4%) genotypes. The protective ε2/ε3 genotype was more common among controls (11.2% vs. 5.1%). Allelic distribution analyses confirmed these patterns. The ε4 allele was markedly more frequent in AD patients than in controls (22.4% vs. 4.5%), whereas ε2 was less frequent in AD (2.6% vs. 7.0%). The ε3 allele remained the most common in both groups, though reduced in AD compared to controls (75.0% vs. 88.4%). The overall difference in allelic frequencies between groups was significant (χ^2^ = 46.1, df = 2, *p* < 0.001).

**TABLE 2:** APOE genotype and allele distribution in controls and AD patients.

Genotype	CTRL	AD
ε2/ε2	3 (1.2)	0 (0.0)
ε2/ε3	27 (11.2)	5 (5.1)
ε2/ε4	1 (0.4)	0 (0.0)
ε3/ε3	191 (78.9)	51 (52.0)
ε3/ε4	19 (7.9)	40 (40.8)
ε4/ε4	1 (0.4)	2 (2.0)

Allele	CTRL	AD
ε2	34 (7.0)	5 (2.6)
ε3	428 (88.4)	147 (75.0)
ε4	22 (4.5)	44 (22.4)

*χ* ^*2*^ = 57.2, df = 5, p < 0.001 ***.

*χ* ^*2*^ = 46.1, df = 2, p < 0.001***.

Values are N (%). Allele counts were derived from genotype data, with each participant contributing two alleles. Thus, allele frequencies represent the total number of ε2, ε3, and ε4 alleles observed in CTRL and AD patients. *p*-values from χ^2^ test. APOE genotyping data were available for 340 participants (242 controls, 98 AD patients). CTRL, cognitively healthy controls; AD, Alzheimer’s disease patients.

### 3.3 Age at natural menopause (ANM) as a predictor of AD risk

Binomial logistic regression analyses were performed to test whether ANM predicted AD risk (Table 3). When entered as a continuous predictor, ANM was significantly associated with AD. In the unadjusted model, each additional year of later menopause increased the odds of AD by 6% (odds ratio [OR] = 1.06, 95% confidence interval [CI] = 1.01–1.11, *p* = 0.019). This effect remained significant after adjusting for education, number of children, BMI, smoking, diabetes, and hypertension (OR = 1.07, 95% CI = 1.02–1.12, *p* = 0.008). None of the covariates included in the adjusted model were significant predictors of AD risk (all *p* > 0.05).

**TABLE 3:** Logistic regression results for age at natural menopause (ANM), reproductive lifespan and AD risk.

Model	OR	95% CI	*p*-value	AUC
*ANM*				
Model 1	1.06	1.01–1.11	0.019*	0.652
Model 2	1.07	1.02–1.12	0.008**	0.681
*Reproductive lifespan*				
Model 1	1.06	1.01–1.11	0.025*	0.653
Model 2	1.07	1.01−1.12	0.015*	0.678

ANM, age at natural menopause. Reproductive lifespan = years between menarche and menopause. OR, odds ratio; CI, confidence interval; AUC, area under the curve. Model 1 = unadjusted; Model 2 = adjusted for education, number of children, body mass index (BMI), smoking status, diabetes, and hypertension. *p < 0.05; **p < 0.01.

### 3.4 Reproductive lifespan as a predictor of AD risk

Binomial logistic regression analyses were performed to test whether reproductive lifespan predicted AD risk (Table 3). When entered as a continuous predictor, reproductive lifespan was significantly associated with AD. In the unadjusted model, each additional year of reproductive lifespan increased the odds of AD by 6% (OR = 1.06, 95% CI = 1.01–1.11, *p* = 0.025). This association remained significant after adjusting for education, number of children, BMI, smoking, diabetes, and hypertension (OR = 1.07, 95% CI = 1.01–1.12, *p* = 0.015). None of the covariates included in the adjusted model were significant predictors of AD risk (all *p* > 0.05).

### 3.5 Moderating role of APOE alleles

**FIGURE 2:**
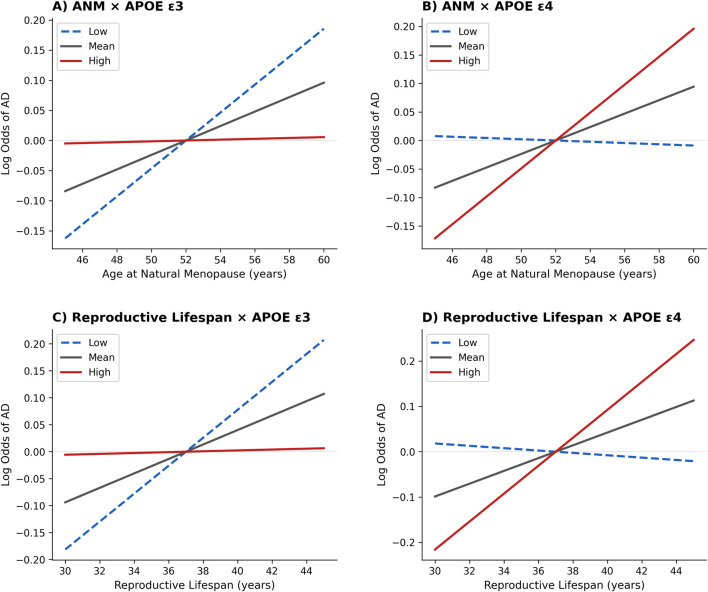
Simple slopes illustrating the interactions between reproductive factors and APOE allele count on sporadic Alzheimer’s disease (AD) risk. Panels display predicted log odds of AD derived from logistic regression moderation models. **(A)** ANM × APOE ε3: later menopause is associated with higher AD risk, with this association progressively attenuated at higher ε3 allele count. **(B)** ANM × APOE ε4: later menopause shows little association with AD at lower ε4 allele count, but a stronger positive association as ε4 allele count increases. **(C)** Reproductive lifespan × APOE ε3: longer reproductive lifespan is associated with higher AD risk, with attenuation at higher ε3 allele count. **(D)** Reproductive lifespan × APOE ε4: longer reproductive lifespan shows a stronger positive association as ε4 allele count increases. Conditional effects are estimated at −1 SD (low), mean, and +1 SD (high) values of allele count. In each panel, the dashed blue line represents low allele count (−1 SD), the solid black line represents the mean allele count, and the solid red line represents high allele count (+1 SD). Lines represent predicted log odds of AD from simple slope analyses. ANM = Age at Natural Menopause; AD = Alzheimer’s Disease.

To test whether reproductive factors influenced the risk of AD differentially according to APOE alleles, three separate moderation models were conducted using the number of ε2, ε3, or ε4 alleles as moderators (Table 4). Across models, both a later ANM and a longer reproductive lifespan were significantly associated with increased AD risk (ANM: β = 0.0117, standard error [SE] = 0.0043, *p* = 0.007; reproductive lifespan: β = 0.0133, SE = 0.0045, *p* = 0.004). Importantly, these effects were significantly moderated by APOE ε3 (ANM: β = −0.0222, SE = 0.0084, *p* = 0.008; reproductive lifespan: β = −0.0249, SE = 0.0089, *p* = 0.005) and APOE ε4 (ANM: β = 0.0308, SE = 0.0105, *p* = 0.003; reproductive lifespan: β = 0.0397, SE = 0.0119, *p* < 0.001), but not by APOE ε2 (ANM: *p* = 0.445; reproductive lifespan: *p* = 0.397). Simple slope analyses clarified the direction of these interactions (Figure 2). In the following simple slope analyses, conditional effects were estimated at low (−1 SD), mean, and high (+1 SD) levels of each moderator. For ε3, given its high prevalence, these levels approximately correspond to fewer than two copies (low), the sample average (mean), and ε3/ε3 homozygosity (high). For ε4, given its low prevalence, low and mean levels both reflect predominantly non-carrier status, while high levels reflect a modest increase in ε4 dosage but do not correspond to ε4/ε4 homozygosity. For ε2, both ANM and reproductive lifespan were positively associated with AD risk at average (ANM: β = 0.0118, *p* = 0.007; reproductive lifespan: β = 0.0131, *p* = 0.004) and low levels (ANM: β = 0.0147, *p* = 0.014; reproductive lifespan: β = 0.0163, *p* = 0.008) of ε2, but not at high levels (ANM: β = 0.0089, *p* = 0.111; reproductive lifespan: β = 0.0098, *p* = 0.083), confirming the absence of moderation. For ε3, the positive association between ANM and AD risk was strongest at low ε3 (β = 0.0232, *p* < 0.001), weaker at average levels (β = 0.0120, *p* = 0.005), and absent at high ε3 (β = 0.0007, p = 0.908); a similar gradient emerged for reproductive lifespan (low ε3: β = 0.0259, *p* < 0.001; mean: β = 0.0134, *p* = 0.002; high ε3: β = 0.0008, *p* = 0.891). This pattern indicates a buffering role of ε3, which mitigates the detrimental impact of prolonged ovarian function on AD vulnerability. In contrast, the effects were amplified among ε4 carriers: for ANM, the slope was negligible at low ε4 (β = −0.0011, *p* = 0.836), modest at average levels (β = 0.0118, *p* = 0.004), and steeply increased at high ε4 (β = 0.0245, *p* < 0.001); for reproductive lifespan, the association was non-significant at low ε4 (β = −0.0026, *p* = 0.660), moderate at average levels (β = 0.0141, *p* = 0.001), and maximal at high ε4 (β = 0.0308, *p* < 0.001). Taken together, these findings demonstrate that both later menopause and extended reproductive lifespan are linked to higher AD risk, but the magnitude of this relationship depends critically on APOE genotype: ε3 attenuates, whereas ε4 amplifies, the adverse effects of prolonged hormonal exposure, revealing a robust allele-specific gene-hormone interaction underlying sex-specific vulnerability to AD.

**TABLE 4:** Moderation effects of APOE alleles on the associations between reproductive factors and AD risk.

Moderator	Predictor	β	SE	*p*-value	Slope (−1 SD)	p-value	Slope (mean)	*p*-value	Slope (+1 SD)	p-value
ε2	ANM	−0.008	0.011	0.445	0.015	0.014	0.012	0.007	0.009	0.111
	Reproductive lifespan	−0.010	0.012	0.397	0.016	0.008	0.013	0.004	0.010	0.083
ε3	ANM	−0.022	0.008	0.008**	0.023	<0.001***	0.012	0.005**	0.001	0.908
	Reproductive lifespan	−0.025	0.009	0.005**	0.026	<0.001***	0.013	0.002**	0.001	0.891
ε4	ANM	0.031	0.011	0.003**	−0.001	0.836	0.012	0.004**	0.025	<0.001***
	Reproductive lifespan	0.040	0.012	<0.001***	−0.003	0.660	0.014	0.001**	0.031	<0.001***

ANM, age at natural menopause. Reproductive lifespan = years between menarche and menopause. β = unstandardized coefficients; SE, standard errors, and *p*-values are reported for moderation and simple slope analyses. Later menopause and longer reproductive lifespan were associated with higher AD risk, with effects significantly moderated by APOE, ε3 and ε4 but not ε2. *p* < 0.05*, *p* < 0.01**, *p* < 0.001***.

## 4 Discussion

This study extends previous research examining reproductive factors and APOE genotype in relation to sporadic AD risk. While prior work, including the Rotterdam study (Geerlings et al., 2001) and the United Kingdom Biobank cohort (Lindseth et al., 2023), has explored this relationship, our work examines dose-dependent moderation effects across all three APOE alleles (ε2, ε3, ε4) in clinically diagnosed AD patients. Three main findings emerged. First, the distribution of APOE genotypes significantly differed between cognitively healthy controls and AD females, with AD patients showing the expected enrichment of ε4 and controls showing relative protection of ε2. Second, both later ANM and longer reproductive lifespan are significantly associated with increased AD vulnerability. Third, these associations are moderated by APOE alleles. Specifically, the detrimental effect of extended ovarian function was amplified among APOE ε4 carriers and attenuated among ε3 carriers, while ε2 showed no significant modulation. Simple slope analyses further revealed a clear allele-dependent gradient, with the steepest increase in AD risk among ε4 carriers, a moderate slope in the general population, and a flat, non-significant pattern among high ε3 carriers. These results identify a previously unrecognized gene-hormone interaction that may contribute to sex-specific susceptibility to AD. Our findings are partially consistent with the Rotterdam study (Geerlings et al., 2001), which reported stronger associations between longer reproductive period and dementia risk among APOE ε4 carriers. However, our study advances this work by demonstrating allele-specific gradient effects across ε2, ε3, and ε4 through simple slope analyses. In contrast to Lindseth et al. (2023), who found no APOE × reproductive factor interactions on cognitive performance in relatively healthy middle-aged females, our focus on clinically diagnosed AD in older patients suggests these gene-hormone interactions may become more pronounced in manifest neurodegenerative disease.

### 4.1 Distribution of APOE genotypes in CTRL and AD groups

Consistent with a large body of literature (Corder et al., 1993; Farrer et al., 1997), our findings confirm that the distribution of APOE genotypes significantly differs between cognitively healthy controls and patients with AD. The ε3/ε3 genotype was the most frequent overall, but its prevalence was markedly higher among controls, whereas AD patients showed a substantial overrepresentation of the ε3/ε4 genotype. Although relatively rare, the ε4/ε4 genotype was almost exclusively observed in the AD group. Conversely, carriers of the ε2/ε3 genotype were more common among controls, in line with the hypothesis of a protective role of ε2. These results are in accordance with previous meta-analyses showing that APOE ε4 is the strongest genetic risk factor for sporadic AD, conferring both an increased likelihood of developing the disease and an earlier age of onset (Corder et al., 1993; Farrer et al., 1997). At the same time, the lower frequency of ε2 among AD patients is consistent with evidence suggesting that this allele may exert a neuroprotective effect (Serrano-Pozo et al., 2015). Importantly, the present data validate the composition of our sample and provide a robust genetic framework for subsequent analyses. Demonstrating the expected enrichment of APOE ε4 in AD patients not only corroborates the validity of our cohort but also sets the stage for exploring how APOE status may interact with reproductive lifespan in modulating dementia risk.

### 4.2 Age at natural menopause (ANM), reproductive lifespan, and dementia risk

Our findings demonstrate that both later ANM and longer reproductive lifespan are significantly associated with an increased risk of AD in females, independent of established clinical and demographic risk factors. Although the two measures were moderately correlated, they capture distinct aspects of endocrine aging: ANM reflects the timing of ovarian senescence, while reproductive lifespan represents the cumulative duration of estrogenic exposure. Considering them jointly allows a more comprehensive understanding of how hormonal trajectories shape late-life neurodegenerative risk. In our sample, females with later menopause showed a significantly higher probability of AD diagnosis, suggesting that prolonged ovarian function - while beneficial for systemic health in midlife - may have adverse consequences for the aging brain. This association was evident in both unadjusted and adjusted models, indicating that menopausal timing and reproductive lifespan exert effects beyond conventional vascular, metabolic, or educational influences. These results contribute to the ongoing debate on the role of cumulative estrogen exposure across the lifespan in modulating cognitive outcomes in females. While some studies suggest that prolonged estrogen exposure may be neuroprotective (Brinton, 2009; Rocca et al., 2011), others have found null or even detrimental associations between longer reproductive span and dementia risk (Georgakis et al., 2016). Notably, Rocca et al. (2011) demonstrated that bilateral oophorectomy resulting in shortened estrogen exposure was associated with increased risk of cognitive impairment and dementia, supporting the protective role of endogenous estrogen. Our simple slope analyses confirmed that both later ANM and longer reproductive lifespan were associated with increased AD risk, following a clear allele-dependent gradient: the slope was steepest among ε4 carriers, moderate in the general population, and flat among females with higher ε3 dosage. Taken together, these findings indicate that later ANM and extended reproductive lifespan are not protective factors; rather, they may contribute to higher AD vulnerability in females, particularly when combined with specific APOE genotypes. This pattern aligns with prior epidemiological evidence linking late menopause and prolonged reproductive periods to greater dementia risk (Georgakis et al., 2016). ANM and reproductive lifespan thus emerge as interrelated but biologically distinct indicators of endocrine aging, both exerting an independent contribution to neurodegenerative susceptibility. Importantly, their effects persisted after adjustment for major covariates, reinforcing the relevance of female-specific hormonal trajectories in models of AD risk.

### 4.3 Moderating role of APOE alleles

The present study provides the first evidence that the relationship between ANM, reproductive lifespan and AD risk is moderated by APOE genotype. While the protective ε2 allele did not interact with ANM and reproductive lifespan, the effects of ε3 and ε4 were markedly different, pointing to allele-specific mechanisms. For APOE ε2, the lack of a significant interaction suggests that its protective influence on AD risk operates independently of reproductive factors. This finding is consistent with prior work attributing to ε2 a generalized neuroprotective role, possibly mediated by reduced amyloid deposition and enhanced synaptic resilience (Serrano-Pozo et al., 2015). Our data imply that this benefit is not contingent on variations in ANM and lifetime estrogen exposure. For APOE ε3, our moderation analyses revealed a buffering effect: the positive association between longer reproductive lifespan and AD was significant only in females with fewer ε3 alleles and was abolished at higher ε3 dosage, indicating a dose-response flattening of the risk curve. This pattern is consistent with prior evidence that ε3 may confer relative protection compared with ε4 and, in some populations, acts as a protective factor against AD rather than a purely “neutral” reference allele (Rebeck et al., 2002; De-Almada et al., 2012). Mechanistically, isoform-specific signaling differences provide a plausible basis for this buffering: compared with ε4, ε3 engages lipid transport and receptor pathways in ways that may better sustain synaptic and neurovascular function (Zhao and Brinton, 2005), thereby attenuating the impact of prolonged estrogenic exposure on downstream neurodegenerative processes. Our findings are consistent with emerging evidence that APOE genotype modifies the relationship between estrogen exposure and dementia risk across multiple study designs and populations. Although associations between estrogen exposure and cognitive outcomes have been inconsistent, a convergent pattern has emerged regarding APOE ε4 as an effect modifier. de Lange et al. (2020) reported that higher estradiol levels were associated with more pronounced brain aging among APOE ε4 carriers in neuroimaging analyses, while Park et al. (2024) demonstrated that the protective association of estrogen exposure with dementia risk was attenuated in APOE ε4 carriers using United Kingdom Biobank data. These epidemiological observations are supported by experimental evidence from Nathan et al. (2004), who demonstrated that estrogen-induced neurite outgrowth occurred synergistically with APOE ε3 but not with APOE ε4 in cultured cortical neurons, revealing genotype-specific differences in estrogen responsiveness at the cellular level. Together with our dose-dependent moderation findings, these converging results from neuroimaging, epidemiological, and experimental studies highlight the critical importance of gene-hormone interactions in dementia pathogenesis and underscore the need for further investigation into how APOE genotype shapes the neurological effects of estrogen exposure across the lifespan.

In line with these converging findings, the most striking result of our study concerns APOE ε4, which not only exerted its well-known main effect as a genetic risk factor, but also significantly amplified the influence of ANM and reproductive lifespan. In females carrying more ε4 alleles, the association between ANM, extended reproductive lifespan and AD risk was markedly stronger, whereas it was negligible in non-carriers. Simple slope decomposition clearly illustrated this amplification: each additional ε4 allele steepened the regression line between reproductive factors and AD risk. This novel gene-environment interaction suggests that ε4 may render the brain more vulnerable to the long-term effects of estrogen exposure. Potential mechanisms include altered estrogen receptor signaling, heightened neuroinflammatory responses, and greater susceptibility to amyloid and tau pathology in the presence of prolonged hormonal influence. This pattern is consistent with the “healthy cell bias” of estrogen action (Brinton, 2008), which posits that estrogens promote neuronal function when acting on healthy cells but may accelerate neurodegenerative processes when the neuronal environment is already compromised - a condition more likely in APOE ε4 carriers, whose neurons exhibit greater baseline vulnerability to oxidative stress and impaired lipid homeostasis. Taken together, these findings reveal a complex and allele-specific interaction between ANM, reproductive lifespan and APOE. While ε2 confers protection irrespective of ANM and reproductive history, ε3 buffers, and ε4 exacerbates, the impact of ANM and prolonged reproductive lifespan on AD risk. This genotype-dependent modulation represents a novel conceptual advance, highlighting ANM and reproductive lifespan not merely as an independent risk factor, but as a variable whose clinical meaning depends critically on APOE status. By integrating the slope analyses, our results delineate a continuum of risk across allelic profiles - from protection (ε2) to buffering (ε3) to amplification (ε4). By uncovering this missing link, our study provides a new framework to interpret the heterogeneity of dementia risk in females and underscores the need for sex- and genotype-specific approaches in both research and prevention.

### 4.4 Limitations and future directions

This study should be interpreted in light of several limitations. First, our study relied on clinical diagnosis of AD based on established NINCDS-ADRDA and NIA-AA criteria (McKhann et al., 1984; 2011). While these criteria are well-validated and widely used in clinical practice and research, biomarker confirmation (e.g., amyloid PET or CSF biomarkers) following current diagnostic frameworks (Jack et al., 2024) would provide greater diagnostic certainty and specificity. Future studies incorporating biomarker data would further strengthen the conclusions regarding the relationship between reproductive factors, APOE genotype, and AD pathology. Second, its retrospective design did not allow for the assessment of temporal or causal relationships between ANM, reproductive lifespan, APOE genotype, and AD risk. Third, reproductive history variables (age at menarche and menopause) were self-reported and may be subject to recall bias, particularly in older participants. For participants with AD, such information was recorded in medical records and, when available, could include caregiver reports or other clinical documentation. However, no independent validation was possible. Fourth, although the sample size was adequate to detect group differences, stratification by APOE alleles resulted in some small subgroups, limiting statistical power for less frequent genotypes (e.g., ε2/ε2 and ε4/ε4). Fifth, the study lacked detailed information on additional reproductive and hormonal factors, such as breastfeeding, use of hormonal contraception, and use of hormone replacement therapy, which could further modulate the association between reproductive lifespan and dementia risk. In particular, hormone replacement therapy may independently influence AD risk and could interact with both APOE genotype and menopausal timing (Yaffe et al., 2000; Kang and Grodstein, 2012), while oral contraceptive use and breastfeeding may alter the cumulative duration and pattern of estrogenic exposure, potentially confounding the estimated effects of reproductive lifespan. The absence of these variables precludes a more fine-grained characterization of lifetime hormonal trajectories. Despite these caveats, the consistency of our slope-based findings across both reproductive variables and allelic groups provides robust internal validity and supports the generalizability of the gene-hormone interaction model. Sixth, although genotype-specific stratified analyses would provide additional granularity, our sample size precludes robust statistical inference for rare genotypes such as ε2/ε2 (n = 3), ε4/ε4 (n = 3), and ε2/ε4 (n = 1). The ε2/ε2 genotype, in particular, has a population frequency below 1%, making adequate representation challenging even in large-scale studies. The allele-count moderation approach we employed is statistically more powerful for detecting dose-dependent effects in samples with this genotypic distribution and is a standard method in genetic epidemiology when rare homozygous genotypes limit stratified analyses. Seventh, our sample consisted exclusively of Italian females of European ancestry, which limits the generalizability of the findings. APOE allele frequencies, reproductive patterns, and AD risk profiles vary substantially across ethnic groups (Farrer et al., 1997), and the gene-hormone interactions observed here may differ in magnitude or direction in non-European populations. Future studies should adopt prospective longitudinal designs, larger and ethnically diverse cohorts, and integrate multimodal biomarkers of AD pathology. In addition, incorporating comprehensive measures of lifetime estrogen exposure and related hormonal factors will be crucial to refine the understanding of how ANM and reproductive history interacts with APOE to shape dementia risk. Ultimately, these efforts may inform precision-prevention strategies tailored to females’ genetic and reproductive profiles.

## 5 Conclusion

In conclusion, this study demonstrates that both ANM and reproductive lifespan are significantly associated with sporadic AD risk in females, and that these relationships are differentially moderated by APOE alleles. While APOE ε2 appears neutral, APOE ε3 exerts a buffering effect, and APOE ε4 amplifies the impact of ANM and longer reproductive lifespan on AD risk. Simple slope analyses clarify that these effects follow a dose-dependent gradient across allelic profiles, defining a novel gene-hormone continuum of risk. These findings provide novel evidence for a genotype-specific interaction between reproductive history and genetic susceptibility, offering a new framework to understand sex-specific vulnerability to dementia. Recognizing ANM and reproductive lifespan as a critical factor, whose clinical significance depends on APOE status, may contribute to advancing personalized prevention strategies in females’ brain health.

## Data Availability

The datasets presented in this article are not readily available because of privacy protection. Requests to access the datasets should be directed to the corresponding author at francesco.bruno@unimercatorum.it; patrizia.spadafora@irib.cnr.it.
